# Targeting Insulin-Like Growth Factor 1 Leads to Amelioration of Inflammatory Demyelinating Disease

**DOI:** 10.1371/journal.pone.0094486

**Published:** 2014-04-09

**Authors:** Matthew F. Cusick, Jane E. Libbey, Nikolaus S. Trede, Robert S. Fujinami

**Affiliations:** 1 Department of Pathology, University of Utah, Salt Lake City, Utah, United States of America; 2 Department of Oncological Sciences and Huntsman Cancer Institute, University of Utah, Salt Lake City, Utah, United States of America; 3 Department of Pediatrics, University of Utah, Salt Lake City, Utah, United States of America; Baylor College of Medicine, United States of America

## Abstract

In patients with multiple sclerosis (MS) and in mice with experimental autoimmune encephalomyelitis (EAE), proliferating autoreactive T cells play an important role in the pathogenesis of the disease. Due to the importance of these myelin-specific T cells, these cells have been therapeutic targets in a variety of treatments. Previously we found that Lenaldekar (LDK), a novel small molecule, could inhibit exacerbations in a preclinical model of MS when given at the start of an EAE exacerbation. In those studies, we found that LDK could inhibit human T cell recall responses and murine myelin responses *in vitro*. In these new studies, we found that LDK could inhibit myelin specific T cell responses through the insulin-like growth factor-1 receptor (IGF-1R) pathway. Alteration of this pathway led to marked reduction in T cell proliferation and expansion. Blocking this pathway could account for the observed decreases in clinical signs and inflammatory demyelinating disease, which was accompanied by axonal preservation. Our data indicate that IGF-1R could be a potential target for new therapies for the treatment of autoimmune diseases where autoreactive T cell expansion is a requisite for disease.

## Introduction

Multiple sclerosis (MS) is the most prevalent autoimmune disease of the central nervous system (CNS) in young adults. This inflammatory demyelinating disease is characterized by a proinflammatory response directed against “self” CNS antigens. A variety of immune cells have been implicated in MS pathogenesis including monocytes/macrophages, activated glial cells and autoreactive T cells [1]–[3]. A preclinical animal model that is used to investigate the role of the immune system and to test the efficacy of new therapies for MS is experimental autoimmune encephalomyelitis (EAE) [4], [5]. EAE can be initiated through the adoptive transfer of myelin-specific T cells or by injection of a variety of CNS proteins or peptides with adjuvants [5]. In EAE, the sex, age and strain of the animal, as well as the protein/peptide used for inoculation, can determine the clinical course and pathologic features of disease [5].

One of the preclinical EAE animal models used to test potential MS therapeutics is the SJL/J mouse sensitized with synthetic myelin peptide(s). Upon injection with the 139–151 peptide of myelin proteolipid protein (PLP_139–151_) with adjuvants, these mice develop relapsing-remitting EAE (RR-EAE), which mirrors the most common disease course in MS patients [6], [7]. EAE in this model results from the major histocompatibility complex (MHC) class II (IA^s^) molecules presenting PLP_139–151_ peptides to the T cell receptor (TCR) on autoreactive CD4^+^ T helper (T_H_) cells. The engagement of the TCR by the peptide-MHC complex is necessary for the activation of the CD4^+^ T cells, which then proliferate and secrete proinflammatory cytokines [8]. The classical models of EAE are due to a T_H_1 or T_H_17 CD4^+^ T cell response to myelin antigens [9].

The testing of a variety of compounds in the EAE animal model has led to the discovery of glatiramer acetate (copaxone), mitoxantrone and natalizumab, which are currently used to treat MS patients [10], [11]. Unfortunately, the current treatment options for this chronic autoimmune disease do not provide a cure for MS, have limited therapeutic benefit to the patient and may have global immunosuppressive properties, thereby leaving a patient susceptible to infection(s) or reactivation of latent virus. Further, current treatments are limited by the specific type of MS, side effects of the drug(s) and/or the cost of treatment. Therefore, new compounds are needed that are capable of suppressing disease, providing neuroprotection and leaving the immune system intact, all beneficial attributes for any new MS therapeutic.

In a recent zebrafish screen of small molecule libraries, we identified a novel compound, 1H-indole-3-carbaldehyde quinolin-8-yl-hydrazone, named Lenaldekar (LDK), capable of inhibiting activated T cells [12]. Initial work on LDK’s effect on human and murine memory T cell responses *in vitro* demonstrated the suppressive nature of LDK for proliferation. Further, the anti-proliferative effect of LDK correlated with the activation level of the T cells that were suppressed in a non-cytolytic manner [13]. The discovery of LDK’s suppressive effect on zebrafish, murine and human T cell proliferation *in vitro* led us to investigate LDK’s mechanism of action by examining the effect of this compound on myelin-specific memory T cell responses in the SJL/J EAE model system. Previously, we found that treatment of mice with RR-EAE at the start of exacerbation limited clinical disease and inflammation [13]. In other studies, Ridges et al [12] found that LDK dephosphorylated members of the phosphatidylinositol 3 kinase (PI3K)/AKT/mammalian target of rapamycin (mTOR) pathway and stalled cells in late mitosis. Here, we extend our findings and provide evidence that LDK inhibits T cell proliferation through its interaction with insulin-like growth factor-1 receptor (IGF-1R). Inflammation, demyelination and axonal damage were monitored at two different time points after LDK treatment. We found decreased inflammation and demyelination at both time points, however, there was an increase in intact axons. T cells isolated from the spleens of LDK-treated mice did not proliferate *ex vivo* compared to T cells isolated from vehicle-treated mice when stimulated with PLP_139–151_, and interleukin (IL)-2 secretion was significantly lower in lymphocytes from the LDK-treated mice compared to lymphocytes from the vehicle-treated mice at higher peptide concentrations. In competition assays, we found that LDK reduced IGF-1R signaling, which is upstream of PI3K/AKT/mTOR, and arrested myelin-specific T cell proliferation. Furthermore, we tested the effect of LDK on the ability of mice to clear virus. C57BL/6 mice clear the neurotropic murine virus, Theiler’s murine encephalomyelitis virus (TMEV). Interestingly, when TMEV-infected C57BL/6 mice were treated with LDK, infected mice were still able to clear viral antigen positive cells, suggesting that, in the CNS, anti-viral immune clearance mechanisms were still intact.

## Methods

### Drug Synthesis

The compound LDK was obtained from the Chembridge DIVERSet library (ChemBridge, San Diego, CA, USA). The compound was resuspended in 100% dimethyl sulfoxide (DMSO) (Sigma, St. Louis, MO, USA) and 40 mg/kg/day of LDK was injected intraperitoneally (i.p.) once a day into mice. An equivalent volume of the vehicle, DMSO, was injected i.p. into mice as a control.

### Animal Experiments

All animal studies were reviewed and approved by the University of Utah Institutional Animal Care and Use Committee (Protocol #12-09006) and conducted in accordance with the guidelines prepared by the Committee on Care and Use of Laboratory Animals, Institute of Laboratory Animals Resources, National Research Council. All efforts were made to minimize suffering. SJL/J female mice (Jackson Laboratory, Bar Harbor, ME, USA) were sensitized at 4–6 weeks of age as previously described [14]. Briefly, mice were injected subcutaneously in the flanks with 200 μl of 1 mM PLP_139–151_ peptide. The emulsion solution was reconstituted complete Freund’s adjuvant, composed of Freund’s incomplete adjuvant (Pierce Biotechnology, Rockford, IL, USA) containing *Mycobacterium tuberculosis* H37 Ra (2 mg/ml) (Difco Laboratories, Detroit, MI, USA), and PLP_139–151_. Mice were intravenously injected with 0.2 μg of *Bordetella pertussis* toxin (List Biological Laboratories, Campbell, CA, USA), in a 100 μl final volume, on days 0 and 2 following sensitization. Mice developed a relapsing-remitting clinical course (RR-EAE). Mice were weighed and scored daily for clinical signs. Clinical scoring was as follows: 0, no clinical disease; 1, loss of tail tonicity; 2, presents with mild hind leg paralysis with no obvious gait disturbance; 3, mild leg paralysis with gait disturbance and paralysis; 4, hind limbs are paralyzed; and 5, moribund or dead. If the mice were paralyzed to the point where they could not feed or groom themselves (moribund), or they lost 20% of their body weight, the mice are euthanized via inhaled anesthetic.

### TMEV Infection

C57BL/6 mice at 5- to 6-weeks of age were obtained from the Jackson Laboratory. Mice were treated i.p. with either LDK (40 mg/kg per mouse) or phosphate-buffered saline (PBS) daily starting at day −1 until day 10 post-infection (p.i.). Mice were anesthetized with isofluorane by inhalation and infected intracerebrally with 3×10^5^ plaque forming units of the Daniels (DA) strain of TMEV or mock infected with PBS at a final volume of 20 μl per mouse. The DA strain of TMEV was propagated as previously described [15]. The mice were observed and weighed daily for 14 and 21 days p.i.

### Immunohistochemistry

Mice were euthanized and perfused with PBS, followed by 4% paraformaldehyde phosphate-buffered solution. Spinal cords and brains were harvested, divided into 12 transverse portions per spinal cord or five coronal slabs per brain, embedded in paraffin and cut into 4 μm thick tissue sections. To visualize myelin, sections were stained with Luxol fast blue. For scoring of spinal cord sections, each spinal cord segment was divided into four quadrants: the anterior funiculus, the posterior funiculus, and each lateral funiculus. Any quadrant containing meningitis, perivascular cuffing or demyelination was given a score of 1 in that pathologic class. The total number of positive quadrants for each pathologic class was determined, then divided by the total number of quadrants present on the slide and multiplied by 100 to give the percent involvement for each pathologic class.

SMI 311 staining was performed on consecutive tissue sections as previously described [16]. Briefly, SMI 311 (Sternberger Monoclonals, Baltimore, MD, USA) is composed of a variety of monoclonal antibodies that recognize non-phosphorylated neurofilament proteins on healthy neurons and dendrites along with damaged axons. Antigen retrieval was performed on the tissue sections by autoclave pretreatment prior to staining with primary antibody at 1∶1000 dilution. After the overnight incubation with primary antibody, tissue sections were washed and incubated for 30 min at room temperature with biotin-conjugated donkey anti-mouse immunoglobulin G antibody (Jackson ImmunoResearch Laboratories, West Grove, PA, USA). After washing, sections were incubated with ABC Vectastain (Vector Laboratories, Burlingame, CA, USA), as per the manufacturer’s recommendations, and visualized with 3,3′-diaminobenzidine tetrahydrochloride (Sigma) in 0.01% hydrogen peroxide (Sigma) in PBS. Counterstaining with Harris hematoxylin (Electron Microscopy Sciences, Hatfield, PA, USA) was performed on these tissue sections. SMI 311 quantification was performed using Image-Pro Plus (Media Cybernetics, Silver Springs, MD, USA). At least six spinal cord tissue slices were imaged per mouse and the data represented is the mean score of the tissue slices quantified.

Silver-staining was performed on tissue sections through the reduction of ammoniacal silver to visible metallic silver. Slides were incubated in pre-warmed (40°C) 10% silver nitrate (Fisher Scientific, Pittsburgh, PA, USA) solution for 15 min and then washed in distilled water. To the silver nitrate solution, concentrated ammonium hydroxide (Fisher Scientific) was added drop by drop until the precipitate formed was just clear. Slides were placed back into this solution and stained at 40°C for 30 min. Slides were placed directly into developer working solution [formaldehyde (Mallinckrodt, Paris, KY, USA), citric acid (trisodium dihydrate, Sigma), concentrated nitric acid (Sigma)] for 30 sec. The reaction was stopped by dipping the slides into 1% ammonium hydroxide solution for 1 min. Sections were washed and incubated in 5% sodium thiosulfate (Fisher Scientific) solution for 5 min. Slides were then washed, dehydrated with alcohol, cleared with xylene, mounted and imaged.

Brain tissue sections from TMEV-infected C57BL/6 mice were stained as previously described [15], [17], [18]. Briefly, DA viral antigen-positive cells were detected on paraffin sections using hyperimmune rabbit serum against TMEV. DA viral antigen-positive cells were enumerated in the following brain regions in C57BL/6 mice: septum, hippocampus, and cortex.

### Ex Vivo Recall Response to PLP_139–151_ in the Presence of Anti-IGF-1R

Spleens were harvested from each mouse on the indicated day post-sensitization. Mononuclear cells were isolated with Histopaque-1083 (Sigma). Cells were resuspended at 1 × 10^6^ cells/ml in complete media [RPMI-1640 media (Mediatech, Manassas, VA, USA) supplemented with 1% L-glutamine (Mediatech), 1% antibiotics (Mediatech), 50 μM 2-mercaptoethanol (Sigma) and 10% Cosmic calf serum (Hyclone, Logan, UT, USA)]. Next, 100 μl of cells were added to each well of a 96-well round-bottomed plate (Corning, Corning, NY, USA). PLP_139–151_ in 100 μl of complete media was added into culture in a dose dependent manner. Cells were incubated at 37°C, 5% CO_2_ for the indicated times in the presence of the indicated peptide doses. Anti-IGF-1R antibody (αIR3) (Millipore, Marlborough, MA, USA) was added into the PLP_139–151_-stimulated spleen cell cultures at a final concentration of 1 μg/ml. Eighteen hrs prior to harvesting cultures, the cells were pulsed with 1 μCi/well of tritiated thymidine (^3^H-TdR) (PerkinElmer, Boston, MA, USA). The cells were harvested onto glass fiber filters (PerkinElmer) for measurement of radiolabel incorporation using a liquid scintillation counter (PerkinElmer).

### IL-2 Enzyme-Linked Immunosorbent Assay (ELISA)

Supernatants were collected at 48 hrs from lymphocytes incubated with PLP_139–151_ peptide, and stored at −80°C until tested. Flat-bottomed 96-well plates (Corning) were coated with anti-IL-2 capture antibody (BD Bioscience, San Jose, CA, USA) overnight at 4°C. Plates were washed, incubated with supernatants, washed and incubated with biotinylated secondary antibody (BD Bioscience). Europium streptavidin (PerkinElmer) was added and plates were developed by using the Delfia Enhancement solution (PerkinElmer). Fluorescence was measured using a Wallac Victor 2 Multi-label Counter (PerkinElmer). Experimental values were determined through the use of a standard curve derived from serial dilutions of a known quantity of recombinant IL-2.

### Statistical Analysis

The program SigmaPlot (Systat Software, Inc., Chicago, IL, USA) was used for all statistical analyses performed. The Student’s paired *t*-test was performed for pairwise comparison.

## Results

### LDK Suppresses ex vivo PLP_139–151_-Specific T Cell Responses

**Figure 1:**
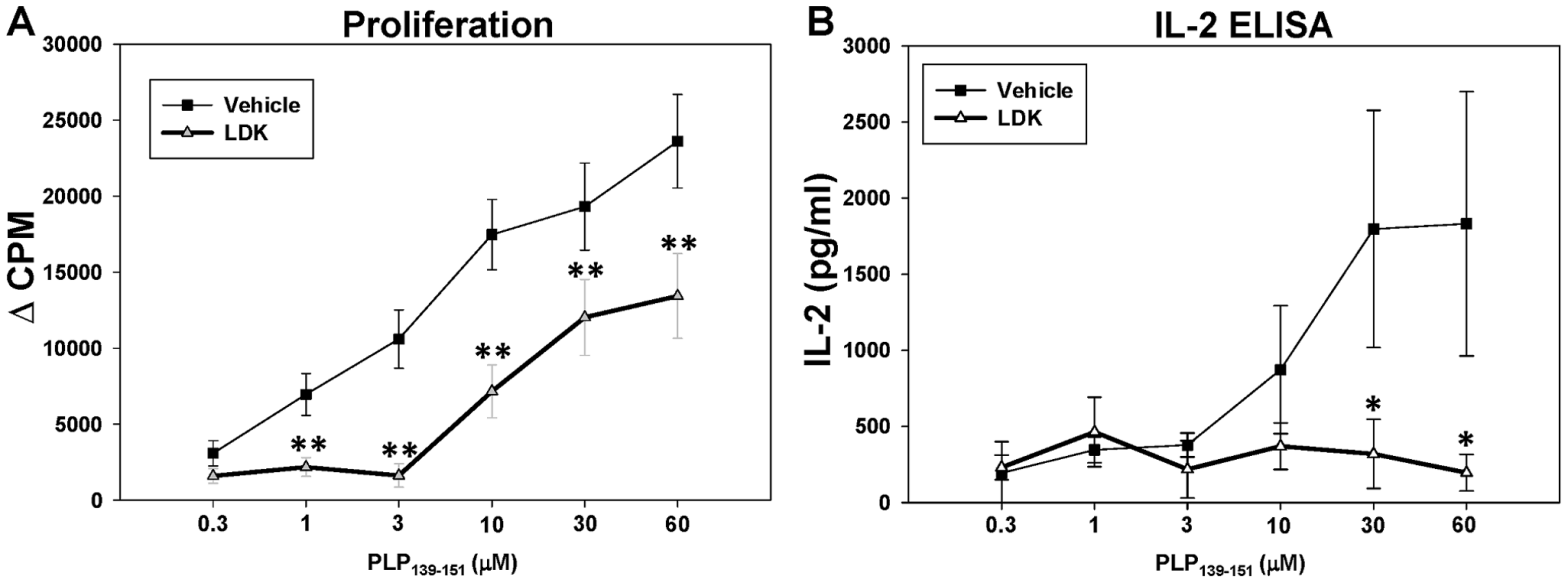
LDK ameliorates EAE through inhibition of PLP_139–151_-specific T cell responses. SJL/J mice were immunized with PLP_139–151_ and treated with either vehicle (DMSO, square) or LDK (40 mg/kg, triangle) once a day for 19 days upon relapse. Spleen cells were then isolated on Day 56 and re-stimulated *ex vivo* with increasing concentrations of PLP_139–151_ peptide. (A) ^3^H-TdR incorporation assays were performed 72 hrs after stimulation with peptide. (B) Cell culture supernatants were collected at 48 hrs post-stimulation with peptide and IL-2 levels were measured by ELISA. Data are representative of the mean ± standard error of the mean (SEM) for groups of 7 mice. Each assay was done in triplicate. *P<0.05, **P<0.005, Student’s paired *t*-test.

The immune cells responsible for EAE in PLP_139–151_-sensitized mice are CD4^+^ T cells. Previously we determined the half maximal inhibitory concentration (IC_50_) for LDK, leading to inhibition of both murine and human T cell proliferation, to be 3 μM [13]. In these studies we sought to determine if LDK had similar effects on PLP_139–151_-specific CD4^+^ T cells obtained directly from LDK-treated animals. Spleens were collected on Day 56 from vehicle- and LDK-treated mice sensitized with PLP_139–151_. This is at a time when vehicle-treated mice were undergoing a second exacerbation (third attack) and the LDK-treated mice showed no signs of clinical disease (data not shown). Lymphocytes were isolated and stimulated *ex vivo* with PLP_139–151_ in a dose-dependent manner (Figure 1). ^3^H-TdR uptake assays were performed 72 hrs after stimulation with peptide (Figure 1A). T cell proliferative assays showed that lymphocytes from the LDK-treated mice had a significantly lower PLP_139–151_ recall response in comparison to lymphocytes from vehicle-treated mice (P<0.005, Student’s paired *t*-test) (Figure 1A).

### LDK Reduces IL-2 Levels

IL-2 is produced by activated T cells. IL-2 can influence certain aspects of antigen-driven T cell responses in an autocrine or paracrine manner. The mTOR and PI3K/AKT pathways are involved. To test for IL-2 production, cell culture supernatants were collected at 48 hrs post-stimulation with different concentrations of PLP_139–151_ and IL-2 levels were measured by ELISA (Figure 1B). The amount of IL-2 secreted from lymphocytes isolated from the LDK-treated mice was similar to the amount of IL-2 secreted from lymphocytes from the vehicle-treated mice at low peptide concentrations, indicating that PLP_139–151_-specific T cells were present in the animals. However, the level of IL-2 secretion was significantly lower in lymphocytes from the LDK-treated mice compared to lymphocytes from the vehicle-treated mice at higher peptide concentrations (P<0.05, Student’s paired *t*-test), suggesting that the activation pathway of the PLP_139–151_-specific T cells was suppressed by LDK (Figure 1B). Interestingly, at lower peptide concentrations the IL-2 secretion was similar between treatment groups, in opposition to what is seen with the proliferative response, suggesting that LDK is not cytolytic but instead modulates CD4^+^ T cells, in that, viable non-proliferating CD4^+^ T cells account for the IL-2 production in the LDK-treated cell cultures stimulated at lower peptide concentrations. Taken together, these results demonstrate that LDK is able to modulate the activation of autoreactive T cells.

### LDK Inhibits Myelin-Specific T Cells by Blocking IGF-1R

**Figure 2:**
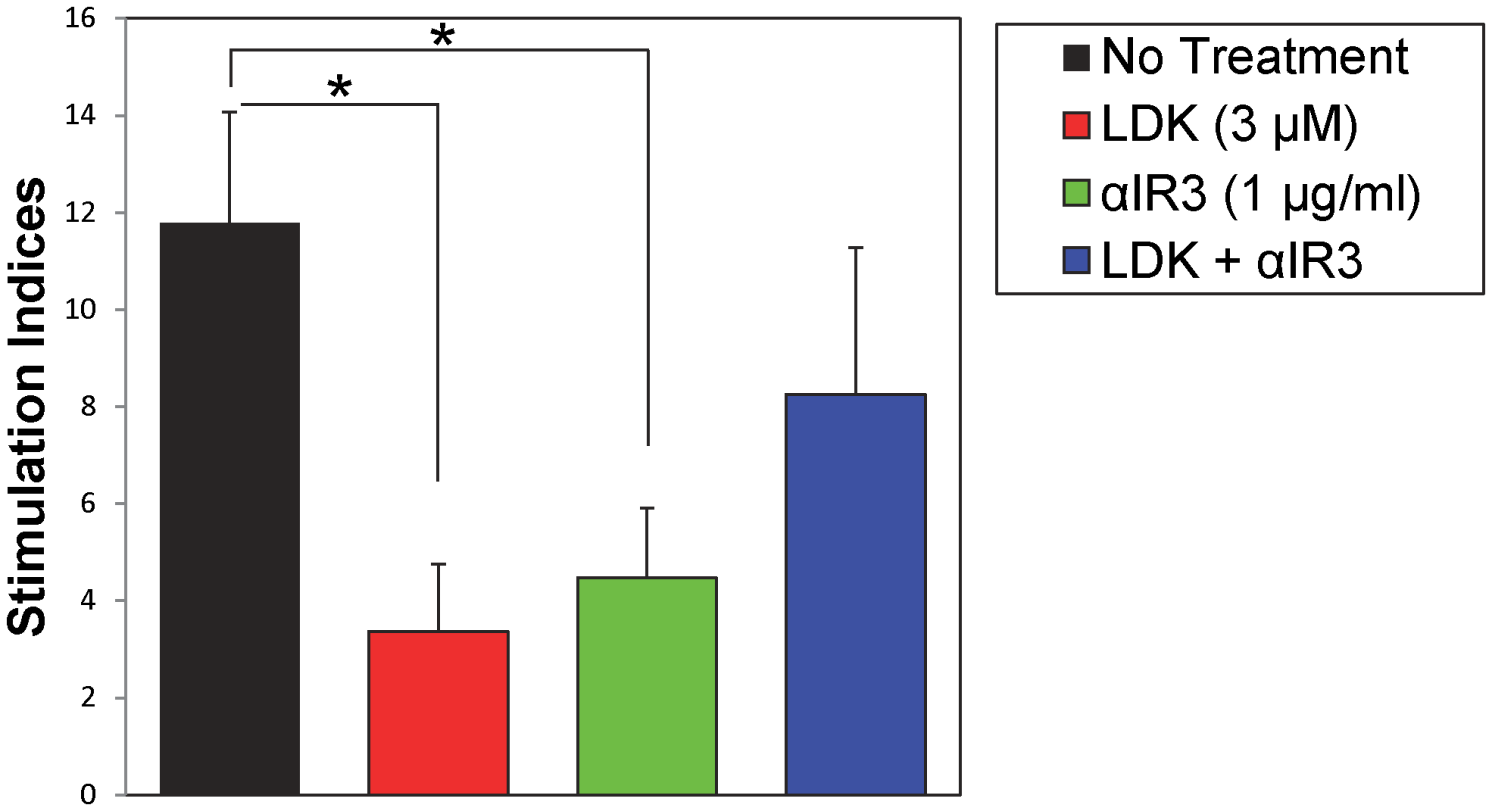
Anti-insulin growth factor receptor 1 (IGF-1R) antibody (αIR3) negates LDK’s suppressive effect. Spleen cells isolated from SJL/J mice immunized with PLP_139–151_ were re-stimulated *ex vivo* with PLP_139–151_ peptide (3 μM). Cells were treated with different combinations of either antibody and/or compound as indicated. ^3^H-TdR incorporation assays were performed 72 hrs after stimulation with peptide. Data are representative of the mean ± SEM for two separate experiments and each assay was done in triplicate for each culture condition. Stimulation index is representative of experimental divided by medium. *P<0.05, Student’s paired *t*-test.

Recently, we have identified one potential target of LDK, IGF-1R (Trede N.S., unpublished data). To determine if LDK was suppressing myelin-specific T cells by blocking IGF-1R, we performed *in vitro* assays using spleen cells isolated from SJL/J mice immunized with PLP_139–151_. Isolated spleen cells were stimulated with PLP_139–151_ peptide (3 μM) and incubated with either anti-IGF-1R antibody (αIR3, 1 μg/ml) and/or LDK (3 μM). Similar to Figure 1A, spleen cells treated with LDK had a significantly lower recall response to the peptide in comparison to untreated spleen cells (Figure 2). In addition, αIR3-treated cultures had a markedly lower T cell response (Figure 2, green bar). However, incubation of cultures with both LDK and αIR3 led to inhibition of the suppressive effect of LDK, resulting in the restoration of T cell proliferation in response to PLP_139–151_ peptide, suggesting that LDK binds to IGF-1R (Figure 2). Taken together, these results demonstrate that αIR3 and LDK neutralize or compete with each other suggesting that both LDK and αIR3 bind to the same or overlapping regions on IGF-1R.

### LDK Reduces Inflammation and Demyelination

**Figure 3:**
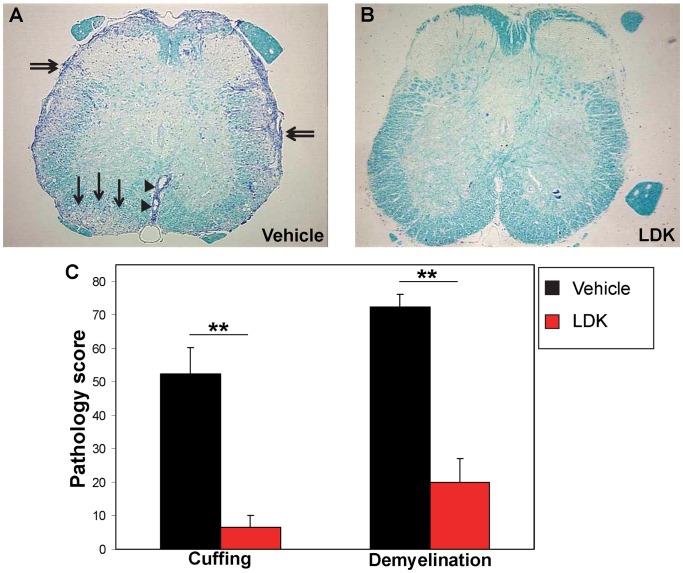
LDK prevents neuroinflammation and demyelination. Spinal cord sections obtained on Day 56 from vehicle-treated (A) and LDK-treated (B) SJL/J mice sensitized with PLP_139–151_ were stained with Luxol fast blue. Infiltration of inflammatory cells in the form of perivascular cuffing (arrowheads) in white matter regions of the spinal cord, meningitis (double arrows) and demyelination (arrows) are indicated. (C) Pathologic scoring of the spinal cord sections was performed as described in the Methods. Data represent the mean pathologic scores+SEM for groups with 7 mice per group. **P<0.005, Student’s paired *t-*test.

Pathological analysis of spinal cord sections from vehicle-treated mice (Figure 3A), sacrificed on Day 56 (during the second exacerbation), showed extensive infiltration of inflammatory cells in the form of perivascular cuffing (arrowheads), meningitis (double arrows) and demyelination (arrows) in comparison to LDK-treated mice (Figure 3B). The LDK-treated mice had statistically lower pathology scores (P<0.005, Student’s paired *t*-test) for perivascular cuffing and demyelination, compared to vehicle-treated mice (Figure 3C). Taken together, these results demonstrate that LDK is able to limit clinical relapses, inflammation and demyelination.

### LDK Limits Axonal Damage

**Figure 4:**
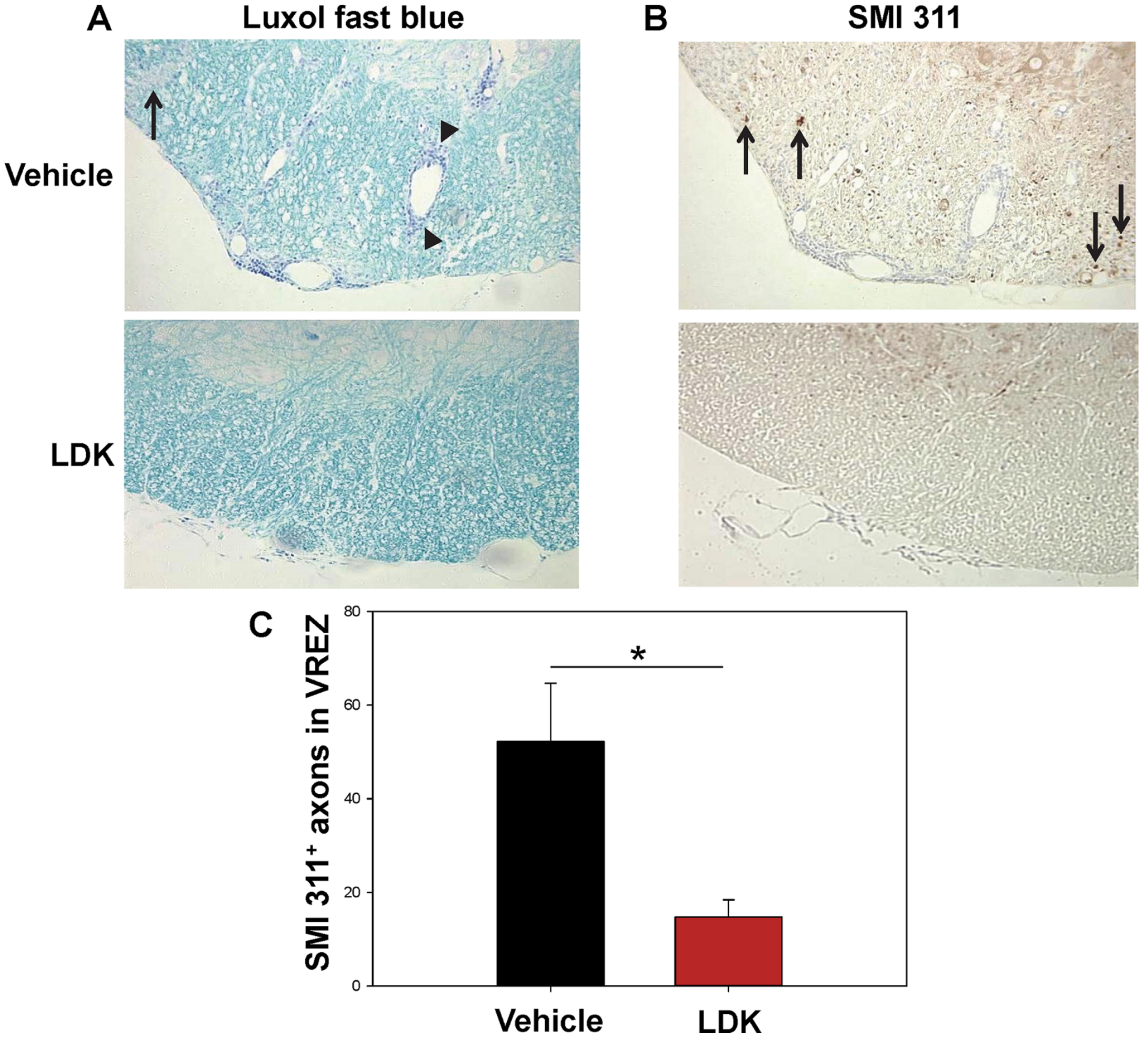
LDK provides axonal preservation during the peak of EAE-relapse. Spinal cord sections obtained on Day 43, the peak of EAE-relapse, from vehicle-treated and LDK-treated SJL/J mice sensitized with PLP_139–151_ were stained with Luxol fast blue and SMI 311. (A) Representative Luxol fast blue stained spinal cord sections showed perivascular cuffing (arrowheads) and demyelination (arrow) in the vehicle-treated mouse in comparison to the LDK-treated mouse. (B) Representative staining of non-phosphorylated neurofilaments using SMI 311 on consecutive tissue sections showed staining in the white matter of the vehicle-treated mouse (arrows), which is indicative of axonal damage. However, SMI 311 is also able to bind to healthy dendrites and nucleated cells in the gray matter. (C) Quantification of SMI 311 positive axons in the white matter of the ventral root exit zone (VREZ) of vehicle-treated and LDK-treated mice at the peak of disease relapse. Results represent the mean+SEM for groups of 3 mice with at least 6 tissue slices per mouse scored by Image Pro-Plus. *P<0.05, Student’s paired *t*-test.

**Figure 5:**
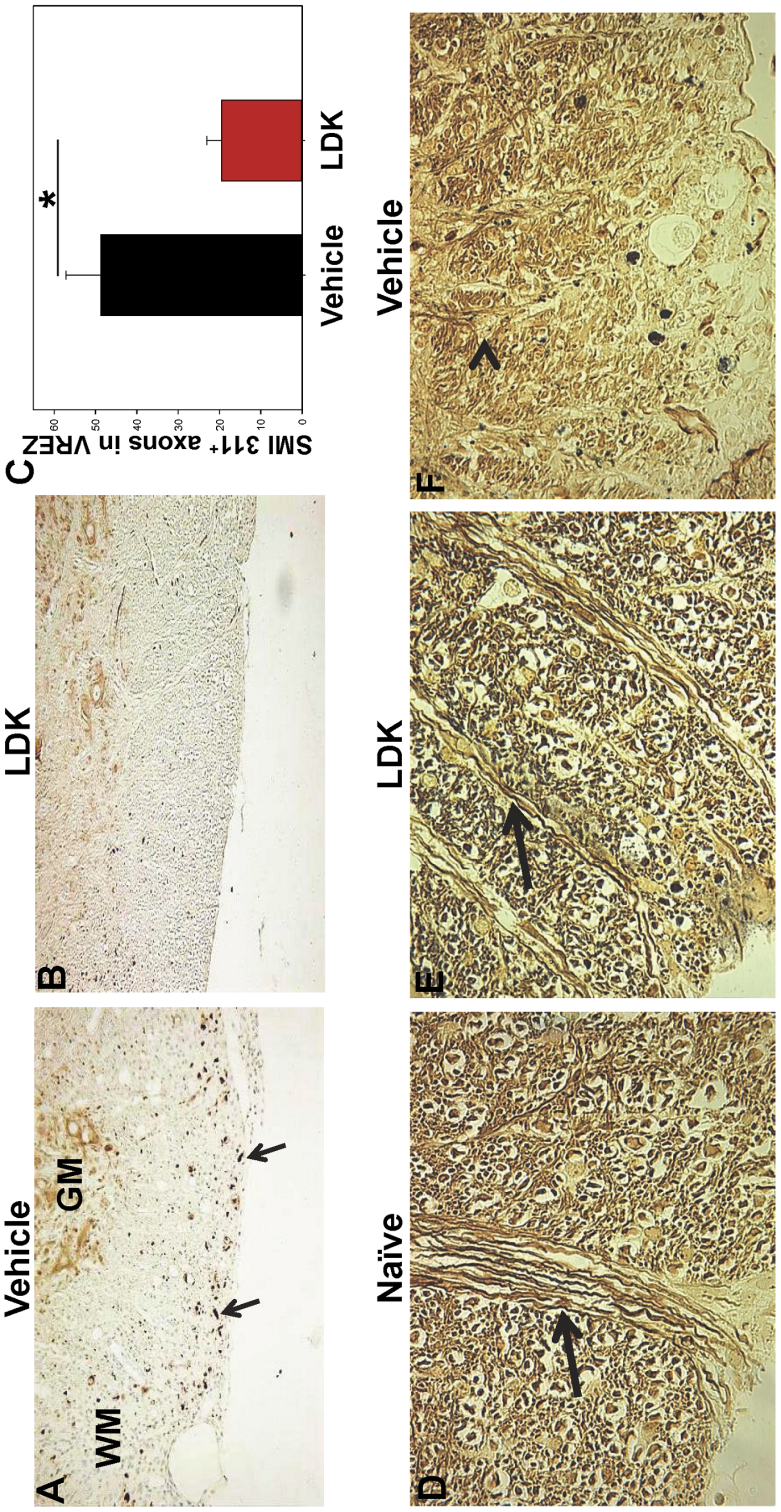
LDK prevents axonal damage in EAE-relapsed mice. Spinal cord sections obtained on Day 56 from vehicle-treated and LDK-treated SJL/J mice sensitized with PLP_139–151_ were stained with SMI 311 and silver stain. (A) Representative spinal cord section of vehicle-treated mouse stained with SMI 311, indicated by the brown dots (arrows). Gray matter (GM) and white matter (WM). (B) Representative spinal cord section of LDK-treated mouse stained with SMI 311. (C) Quantification of SMI 311 positive axons in the white matter of the VREZ. Data is representative of the mean+SEM for groups of 7 mice with at least 5 tissue slices per mouse scored by Image Pro-Plus. *P<0.05, Student’s paired *t*-test. (D–F) Representative silver-stained spinal cord tissue sections of Naïve (D) SJL/J mice, used as a control, and SJL/J mice sensitized with PLP_139–151_ peptide and treated with either LDK (E) or vehicle (F). Intact axons (arrow) and blebs (arrowhead) are indicated. Images are 60× magnification of VREZ.

One of the pathological features of MS is axonal damage due to activated T cells and macrophages/microglial cells. To investigate whether LDK was able to protect mice with EAE from axonal damage, spinal cord sections were stained with SMI 311 which recognizes non-phosphorylated neurofilament proteins. SMI 311 can be used to detect damaged axons in the white matter and healthy dendrites and cell bodies in the gray matter (reviewed in [19]). Mouse spinal cord tissue sections were stained and analyzed from both LDK-treated and vehicle-treated groups at the peak of disease (Figure 4, Day 43) and at the end-point of this study when the vehicle-treated mice had recovered from the first EAE-relapse and were experiencing a second EAE-relapse (Figure 5, Day 56). Luxol fast blue staining of spinal cord tissue sections from vehicle-treated mice at the peak of EAE-relapse (Day 43) showed extensive perivascular cuffing (arrowheads) and demyelination (arrow), compared to LDK-treated mice (Figure 4A). SMI 311 staining of consecutive sections (Figure 4B) and quantification of SMI 311 positive axons in the ventral root exit zone (VREZ) (Figure 4C) showed that vehicle-treated mice at the peak of EAE-relapse (Day 43) had a significantly higher number of SMI 311 positive axons (damaged) (Figure 4B, arrows) in comparison to LDK-treated mice (P<0.05, Student’s paired *t*-test). Perivascular cuffing in the vehicle-treated mice (Figure 4A) did not correlate with SMI 311 staining in consecutive sections (Figure 4B), suggesting that SMI 311 staining was not a false positive due to cell infiltration. Further, SMI 311 staining of spinal cord tissue sections from vehicle-treated (Figure 5A) and LDK-treated (Figure 5B) mice and quantification of SMI 311 positive axons in the VREZ (Figure 5C) at Day 56 showed that vehicle-treated mice had a significantly higher number of SMI 311 positive axons (Figure 5A, arrows) in comparison to LDK-treated mice (P<0.05, Student’s paired *t*-test). To further assess the ability of LDK to preserve axons, consecutive spinal cord tissue sections were silver-stained (Figure 5D–F). Representative silver-stained spinal cord tissue sections of the VREZ demonstrated axonal preservation in LDK-treated mice in comparison to vehicle-treated mice (Figure 5D–F). Therefore, LDK was able to limit axonal damage both at the peak of disease (Figure 4, Day 43) and at the end-point of this study when the vehicle-treated mice had recovered from the first EAE-relapse and were experiencing a second EAE-relapse (Figure 5, Day 56), suggesting that administration of LDK upon EAE-relapse provided axonal preservation.

### Neurotropic Virus-Infected LDK-Treated Mice Still Clear Virus-Infected Cells

**Figure 6:**
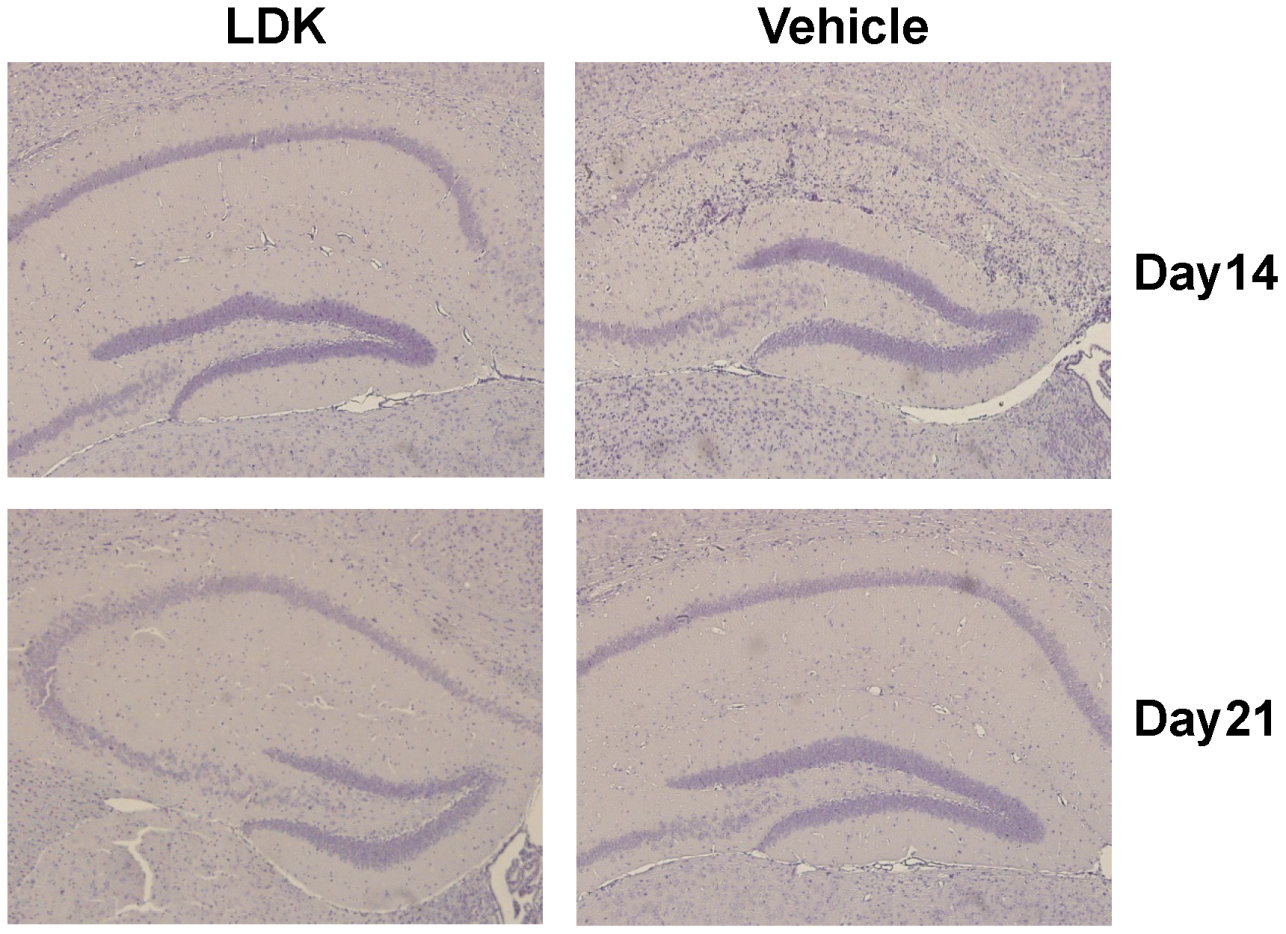
LDK treatment does not affect the ability of C57BL/6 mice to clear TMEV. C57BL/6 mice were treated with either vehicle or LDK starting at day −1 p.i. and continuing until day 10 p.i. Immunohistochemistry using hyperimmune rabbit serum against TMEV was used to detect TMEV antigen positive cells which are normally visible in pyramidal neurons of the hippocampus at day 7 p.i. in mice with seizures [18], but the viral antigen is cleared by day 14 p.i. in TMEV-infected C57BL/6 mice. Images are representative of TMEV-infected mice, at the indicated time points, treated with LDK or vehicle.

Due to LDK’s suppressive effects on immune cells and the role that immune cells play in the clearance of virus, we examined whether LDK-treated C57BL/6 mice were able to clear a neurotropic virus. C57BL/6 mice were treated with either vehicle or LDK (40 mg/kg per day) starting one day prior to infection and continuing for ten days after infection. Mice were infected with TMEV and observed daily. Brain tissue sections were obtained at both 14 (10 mice per group) and 21 (8 mice per group) days p.i. and stained for TMEV antigen. No differences were found in the amount of TMEV antigen detected when comparing vehicle- and LDK-treated mice (Figure 6), suggesting that LDK treatment does not suppress the host immune response to a pathogen.

## Discussion

Recently, we found that LDK binds to IGF-1R (Trede N.S., unpublished data). In addition, we found that LDK was able to suppress EAE exacerbations in a preclinical model for MS as well as inhibit T cell proliferation to both mitogen and specific antigens [13]. In this current study, investigating LDK’s mechanism of action, we found that LDK could inhibit T cell responses to myelin *in vivo*. LDK’s ability to suppress cellular infiltration, in the form of perivascular cuffing, into the spinal cords of PLP_139–151_-sensitized mice *in vivo* (Figure 3) and to suppress recall responses, in the form of T cell proliferation and IL-2 production, to the self-peptide *ex vivo* (Figure 1) suggests that the compound is able to prevent demyelination (Figure 3) by inhibiting autoreactive T cells. Alteration of the IGF-1 signaling pathway could explain the marked reduction seen in both T cell proliferation and inflammation. Thus, we have demonstrated that LDK was able to ameliorate the pathological damage of myelin-specific T cells by potentially targeting IGF-1R on T cells.

Axonal damage is present in the white matter lesions of the spinal cords of EAE mice and in the white matter lesions of the brains of MS patients [3]. We found that in addition to LDK’s ability to reduce two important indicators of inflammation: perivascular cuffing and demyelination (Figures 3, 4A), LDK treatment was also able to prevent axonal damage in the white matter of the spinal cord (Figures 4B, C, and 5), which was likely due to a reduction in inflammation or possible through a direct protective effect. The number of SMI 311 positive axons in spinal cord sections from vehicle-treated mice at the peak of disease was significantly greater than the amount found in sections from LDK-treated mice (Figure 4C), indicating LDK was able to preserve axons. As a means of determining whether the reduction in axonal damage was a consequence of a reduced inflammatory response or of a direct effect of LDK on axons and neurons, we compared the Luxol fast blue (Figure 4A) and SMI 311 (Figure 4B) stained spinal cord sections from vehicle-treated mice. The comparison between inflammation and axonal damage clearly shows that there is no correlation between SMI 311 staining (axonal damage) [19] and perivascular cuffing (inflammation) [14], [20]. Similarly, silver-staining demonstrated that LDK-treated EAE mice had intact axons, as opposed to vehicle-treated mice (Figure 5D-F), thereby supporting the SMI 311 results. Therefore, since axonal damage is not associated with inflammation, LDK could potentially have a direct effect on axons and/or neurons in addition to LDK’s anti-inflammatory properties. However, further studies are needed to determine the mechanism(s) by which LDK is potentially acting directly on neurons, thereby leading to neuroprotection.

Glatiramer acetate therapy is used in RR-MS and is also believed to have a dual mechanism of action [21]. It has been found to have not only immunomodulatory effects, shifting the immune response from a T_H_1 response to a T_H_2 response [5], [22], but the compound can directly stimulate neurotropin, which is involved in neuronal repair [21], [22]. It should be noted that glatiramer acetate has been approved for treatment for over a decade; however the mechanism of how this compound works is currently unknown.

Previous work characterizing LDK’s potential target(s) demonstrated that LDK inhibited the PI3K/AKT/mTOR pathway via an indirect mechanism [12]. Recently, we have identified one potential target of LDK upstream of this pathway, which is IGF-1R (Trede N.S., unpublished data). IGF-1R is a transmembrane receptor tyrosine kinase. A variety of cell types express IGF-1R including oligodendrocyte precursor cells (OPCs) and T cells. IGF-1 signaling through IGF-1R has been demonstrated to be important for oligodendrocyte survival, myelination and proliferation *in vitro*, which suggests a role for IGF-1 and IGF-1R in myelination in the CNS [23]–[25]. Due to the importance of IGF-1 for oligodendrocyte differentiation and survival, numerous studies have tested IGF-1’s efficacy in EAE rodent models. Although some studies had shown that IGF-1 was able to slightly improve remyelination during the acute phase of EAE; these studies found no significant difference between IGF-1 treated and untreated EAE mice at later time points [26]–[28]. Largely based upon the early EAE results, a clinical study was performed in which recombinant human IGF-1 was administered to seven MS patients; however it resulted in no clinical benefit [29]. Furthermore, these studies found that IGF-1 could have potentially led to enhanced myelin-specific T cell responses and exacerbated EAE [27], [30]. Taken together, these studies suggest that IGF-1R activation on T cells could potentially enhance encephalitogenic T cell proliferation, potentially causing exacerbation of disease. Our data are not at odds with these earlier findings in that we are likely altering receptor signaling rather than enhancing signaling through this pathway, as occurs with the addition of IGF-1.

Previous studies have suggested that targeting of the AKT/mTOR pathway could be a viable approach for the inhibition of MS. For example, AKT is a widely expressed protein in many cell types, and has an essential role in a variety of cellular processes including cell proliferation, apoptosis, nitric oxide-mediated events, and glucose metabolism [31]. The PI3K/AKT pathway can be activated through cross-linking of B and T cell receptors, integrins, cytokine receptors and other stimuli [32]–[34]. An important downstream signaling protein of AKT is mTOR [35]. Recent studies of EAE models in both Dark Agouti rats and SJL/J mice have demonstrated that rapamycin, an inhibitor of mTOR, not only ameliorates EAE, but leads to an expansion of natural regulatory T cells [36]. Compelling genetic evidence using microarray data analysis of samples of human white matter obtained from a cohort of healthy and MS post-mortem brains found an upregulation of genes in the PI3K/AKT axis, indicating that this signaling pathway is important in MS [37]. Due to the central importance of the PI3K/AKT pathway in a number of cellular processes, targeting this signaling complex could have negative effects on certain cell types, especially in the CNS. However, Paintlia et al. [38] had documented that statins (lovastatin) promoted the differentiation of OPCs and remyelination in EAE models. Further, *in vitro* experiments showed that lovastatin treatment of OPCs was associated with PTEN (Phosphatase and Tensin Homologue Deleted from Chromosome-10) induction and subsequent inhibition of the PI3K/AKT pathway, which in turn induced cell cycle arrest at the G1 phase without promoting apoptosis [38]. Therefore, these studies suggest that targeting the PI3K/AKT/mTOR pathway is not necessarily detrimental to cell types in the CNS and could possibly promote neuronal repair. However, it is not known how IGF-1R and its signaling axis impacts CD4^+^ T cell differentiation into different CD4^+^ T cell subsets and the progression of EAE/MS and further investigation into IGF-1R on CD4^+^ T cell differentiation is currently under investigation.

An immunosuppressive drug regimen used to treat an autoimmune disease can leave a patient susceptible to infection and/or reactivation of a latent virus. For example, rituximab, a monoclonal antibody directed at CD20, depletes B cells; however, this treatment has led to reactivation of viral infections, such as hepatitis B, cytomegalovirus (CMV), and varicella-zoster virus, in non-Hodgkin lymphoma patients and rheumatoid arthritis (reviewed in [39]). In this study, we showed that LDK-treated mice were still able to clear a neurotropic virus (Figure 6). Recently, it has been hypothesized that mTOR inhibitors, sirolimus and tacrolimus, are able to reduce the incidence of CMV by inhibition of the mTOR pathway (reviewed in [40]). This could be due to CMV’s dependence on mTORC1 for late viral replication in macrophages. In addition, numerous studies have demonstrated that rapamycin treatment could increase the generation of CD8^+^ memory T cells (reviewed in [41]). Thus, targeting the mTOR pathway is a viable option to dampen an autoimmune response while leaving the immune system intact to fight viral infections.

In summary, in the current study we tested the efficacy of LDK treatment in a RR-EAE model of MS to determine if this compound was able to ameliorate an autoimmune response that had already been initiated. We showed that LDK was able to ablate EAE-relapse *in vivo*. LDK inhibited neuroinflammation at the peak of EAE-relapse and at the end-point of this study, demonstrating LDK’s potency at various time points in an autoimmune response. Importantly, LDK-treated mice had significantly less axonal damage in the spinal cord, as indicated by SMI 311 and silver-staining, in comparison to naïve and vehicle-treated mice, thereby demonstrating that LDK provides axonal preservation through inhibition of IGF-1R signaling in T cells. Also, LDK-treated mice were still able to clear virus. Taken together, LDK appears to be an excellent candidate compound to treat immune-mediated diseases.
